# Analgesic Effect of Human Placenta Hydrolysate on CFA-Induced Inflammatory Pain in Mice

**DOI:** 10.3390/ph17091179

**Published:** 2024-09-07

**Authors:** Keun-Tae Park, Heejoon Jo, So-Hyun Jeon, Kyeongsoo Jeong, Minju Im, Jae-Won Kim, Jong-Pil Jung, Hoe Chang Jung, Jae hun Lee, Woojin Kim

**Affiliations:** 1 Department of Physiology, College of Korean Medicine, Kyung Hee University, Seoul 02453, Republic of Korea; cerex@naver.com (K.-T.P.); hjjo@khu.ac.kr (H.J.); 2 Korean Medicine-Based Drug Repositioning Cancer Research Center, College of Korean Medicine, Kyung Hee University, Seoul 02453, Republic of Korea; 3 Research and Development Center, Green Cross Wellbeing Corporation, Yongin 16950, Republic of Korea; sh.jeon@gccorp.com (S.-H.J.); ksjeong@gccorp.com (K.J.); minjulim@gccorp.com (M.I.); kimphage@gccorp.com (J.-W.K.); 4 Nuke Medical Society of Pain Research, Daejeon 35002, Republic of Korea; arinedr@nate.com (J.-P.J.); beriyth@naver.com (H.C.J.); osdoctor1977@naver.com (J.h.L.)

**Keywords:** human placenta hydrolysate, inflammatory pain, pro-inflammatory cytokine, glial cell

## Abstract

To evaluate the efficacy of human placenta hydrolysate (HPH) in a mice model of CFA-induced inflammatory pain. TNF-α, IL-1β, and IL-6 are key pro-inflammatory cytokine factors for relieving inflammatory pain. Therefore, this study investigates whether HPH suppresses CFA-induced pain and attenuates the inflammatory process by regulating cytokines. In addition, the relationship between neuropathic pain and HPH was established by staining GFAP and Iba-1 in mice spinal cord tissues. This study was conducted for a total of day 28, and inflammatory pain was induced in mice by injecting CFA into the right paw at day 0 and day 14, respectively. 100 μL of 20% glucose and polydeoxyribonucleotide (PDRN) and 100, 200, and 300 μL of HPH were administered intraperitoneally twice a week. In the CFA-induced group, cold and mechanical allodynia and pro-inflammatory cytokine factors in the spinal cord and plantar tissue were significantly increased. The five groups of drugs evenly reduced pain and gene expression of inflammatory factors, and particularly excellent effects were confirmed in the HPH 200 and 300 groups. Meanwhile, the expression of GFAP and Iba-1 in the spinal cord was increased by CFA administration but decreased by HPH administration, which was confirmed to suppress damage to peripheral ganglia. The present study suggests that HPH attenuates CFA-induced inflammatory pain through inhibition of pro-inflammatory cytokine factors and protection of peripheral nerves.

## 1. Introduction

Pain, particularly inflammatory pain, is a major global health concern [1]. Inflammation often leads to pain through processes that can result in allodynia—a heightened sensitivity to stimuli such as chemicals, heat, and mechanical pressure [2,3]. Inflammatory pain is typically associated with a widespread state of inflammation, which is a response to tissue damage from various causes, including tumors, trauma, infections, and immune responses. This process involves both tissue defense and subsequent healing [4].

Tissue damage or inflammatory stimuli lead to the release of cytokines, which are crucial in the development of inflammatory pain. Pro-inflammatory cytokines like interleukin 1β (IL-1β) and tumor necrosis factor-alpha (TNF-α) have been shown to lower the threshold for thermal or mechanical allodynia in animal models [5,6,7]. Several studies have reported a correlation between cytokine levels in tissues and the experience of hyperalgesia and pain [8,9]. In response to external infection, injury, and inflammation, glial cells become activated and release pro-inflammatory cytokines, such as TNF-α, IL-6, and IL-1β [10,11,12]. These cytokines are key players in neuroinflammation, serving as signaling molecules in the immune response. The activated neurons, in turn, receive signals from glial cells, perpetuating long-term inflammation and increased pain sensitivity [13]. While the role of cytokines in inflammatory pathways is established, the precise mechanisms that regulate their production and release remain unclear. Research indicates that astrocytes and microglia, non-neuronal cells in the central nervous system (CNS), play a significant role in regulating pain by supporting overall nerve function [14,15].

Most analgesic drugs used until recently are anticonvulsants, non-steroidal anti-inflammatory drugs, and opioids, but their effectiveness in treating chronic pain is limited and may be accompanied by side effects [16]. Therefore, it is essential to discover the specific factor mechanisms that underlie the onset and persistence of chronic pain with multiple pathological causes.

Human placenta hydrolysate (HPH), also known as placenta hydrolysate, is derived from the placenta after delivery. HPH contains high levels of growth factors, essential amino acids and nucleic acids, and anti-inflammatory peptides that promote tissue regeneration [17,18]. Studies on various biological functions of HPH have been reported. Several studies using animal models have reported improvements in liver function [19,20], anti-inflammation [21], platelet aggregation activity [22], glucocorticoid inhibition ability [23], and corticotropin-releasing factor (CRF)-like effects that affect the expression of IL-8 [24]. HPH was shown to relieve symptoms and have an antinociceptive effect on allodynia in a CFA-induced mice chronic arthritis model study [21]. Clinical trials in which HPH was injected subcutaneously improved various inflammation-related symptoms of complex regional pain syndrome, along with anti-inflammatory effects on pain disorders [25,26]. Additionally, in patients with osteoarthritis, HPH injection also reduced knee joint swelling and alleviated pain, thereby improving workers’ daily working hours [27]. Therefore, in this study, we hypothesized that HPH could suppress pain caused by CFA and attenuate the inflammatory process by suppressing the production of pro-inflammatory cytokines mediated by immune responses. Additionally, we aimed to confirm that CFA-induced glial cell damage in the spinal cord was alleviated by HPH, thereby confirming its potential as a preventive agent for chronic inflammatory pain.

## 2. Results

### 2.1. Antinociceptive Effect of HPH in CFA-Induced Cold, Mechanical Allodynia

**Figure 1:**
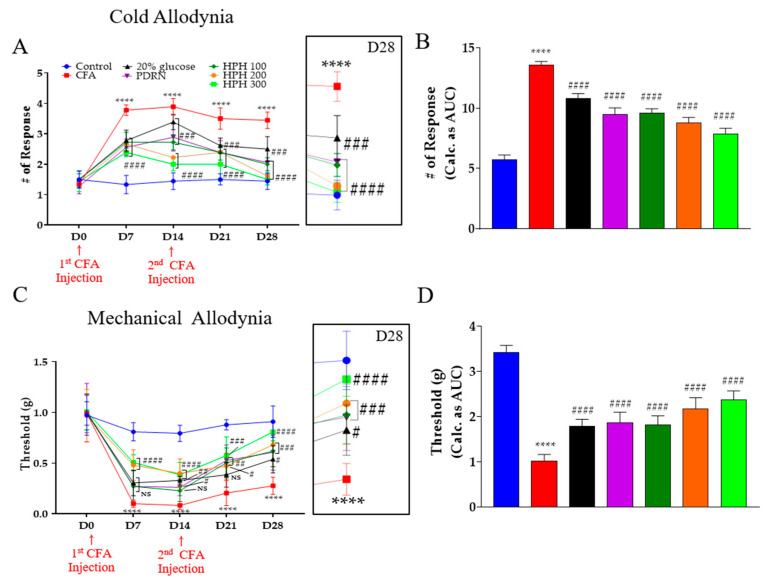
Effects of intraperitoneal administration of 20% glucose, PDRN, and HPH on cold and mechanical allodynia induced by CFA-induced inflammatory pain. Cold (**A**) and mechanical (**C**) allodynia and time courses in the area under the curve (**B**,**D**) of 20% glucose, PDRN, HPH 100, HPH 200, HPH 300 on CFA–induced inflammatory. CFA, complete Freund’s adjuvant; PDRN, polydeoxyribonucleotide; HPH, human placenta hydrolysate, *n* = 6 in each group. Data are expressed as the mean ± SD. NS, not significant. **** *p* < 0.0001 vs. control, # *p* < 0.05, ## *p* < 0.01, ### *p* < 0.001, #### *p* < 0.0001 vs. CFA with two-way analysis of variance followed by Tukey’s post-test for multiple comparisons.

To test the antinociceptive effect of HPH on CFA-induced inflammatory pain, we analyzed its effect on cold and mechanical allodynia (Figure 1). Complete Freund’s adjuvant (CFA) is an immunomodulatory agent that maintains a rapid, strong, and prolonged immune response, making it highly reactive [28,29], and it is used as an inflammatory agent in animal experiments [30,31]. In this study, polydeoxyribonucleotide (PDRN) and 20% glucose were used as positive controls. PDRN has been reported to have positive therapeutic effects in reducing anti-inflammatory symptoms and pain in numerous clinical [32] and animal model studies [33]. Glucose injection therapy is also used clinically to reduce neuropathic pain [34] and is widely applied in clinical practice [35].

All five drug groups demonstrated significant antinociceptive effects from day 7 (D7) to day 28 (D28). On day 28, the effectiveness of the drugs in producing antinociceptive effects was ranked as follows: 20% glucose < PDRN ≤ HPH 100 < HPH 200 ≤ HPH 300 (Figure 1A). When analyzing the antinociceptive effects on mechanical allodynia, only the HPH 200 and HPH 300 groups showed significant effects on day 7 (D7). By day 14 (D14), all drug groups except HPH 100 demonstrated significant antinociceptive effects (Figure 1B). However, by day 28 (D28), all five drugs significantly alleviated pain, with the order of effectiveness confirmed as 20% glucose < PDRN ≤ HPH 100 < HPH 200 < HPH 300.

### 2.2. HPH Decreases Paw Thickness and Pro-Inflammatory Cytokines Increased by CFA Injection

Quantitative polymerase chain reaction (qPCR) was performed to analyze the anti-edema effect and pro-inflammatory cytokine levels of five drugs in the paws of mice. Local edema occurs when blood vessels in the inflamed area relax, capillary permeability, and blood flow increases [36]. This state of increased vascular permeability is induced by the administration of CFA [37].

**Figure 2:**
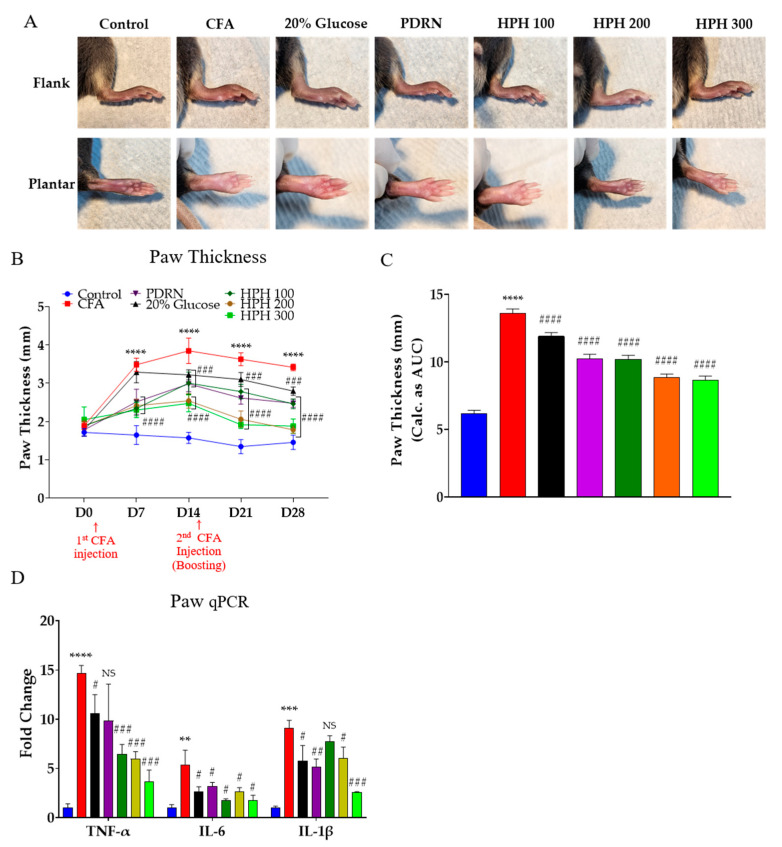
Microscopic view and paw edema measurement and qPCR of pro-inflammatory cytokine factor in mice paw. Effects of intraperitoneal administration of 20% glucose, PDRN, HPH 100, HPH 200, and HPH 300 on the inflammatory response at D14 after CFA-induced paw edema (**A**). The thickness of paw was measured at D0, D7, D14, D21, and D28 (**B**) and time courses in the area under the curve (**C**). qPCR of pro-inflammatory cytokine factor in mice paw (**D**). CFA, complete Freund’s adjuvant; PDRN, polydeoxyribonucleotide; HPH, human placenta hydrolysate, *n* = 6 in each group. Data are expressed as the mean ± SD. NS, not significant. **** *p* < 0.0001, *** *p* < 0.001, ** *p* < 0.01 vs. control, # *p* < 0.05, ## *p* < 0.01, ### *p* < 0.001, #### *p* < 0.0001 vs. CFA with one-way analysis of variance followed by Tukey’s multiple comparisons.

Analyzing the anti-edema effect, paw thickness was significantly reduced in all drug groups except for the 20% glucose group on day 7 (D7). By day 28 (D28), paw thickness was significantly reduced in all five drug groups (Figure 2A,B). The effectiveness of the anti-edema drugs was ranked as follows: 20% glucose < PDRN ≤ HPH 100 < HPH 300 ≤ HPH 200. Figure 2A is a representative image of D14 when the mice paw was the thickest due to CFA injection. Gene expression analysis was conducted on three pro-inflammatory cytokine target genes in mice paw tissues that developed edema due to CFA injection. The qPCR analysis of TNF-α, IL-6, and IL-1β in the mice paws showed that expression levels were significantly increased by approximately 5 to 14 times in the CFA group. The expression levels of these three factors were significantly decreased in the drug-treated groups compared to the control group (Figure 2C).

### 2.3. Ameliorating Effect of HPH on CFA-Induced Histological Damage

**Figure 3:**
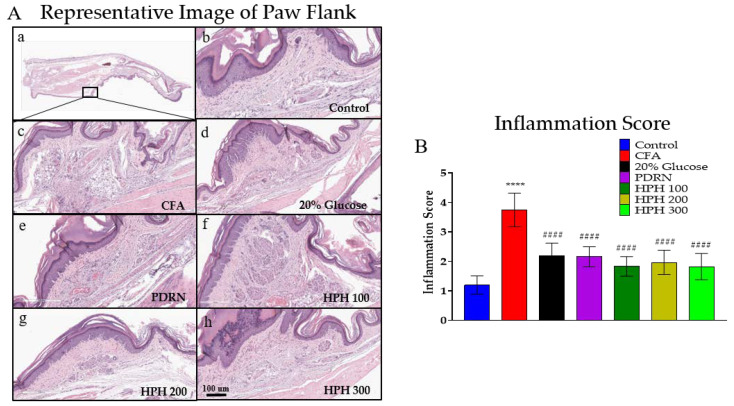
Microscopic examination of H&E stained sections of mice hind paw and inflammation scoring. Representative images show the degree of inflammation in the paws of mice stained with H&E (**A**), with the levels of inflammation scored (**B**). The panels represent (**a**) a representative image of H&E staining of the mice paw, (**b**) the control group, (**c**) CFA group, (**d**) 20% glucose group, (**e**) PDRN group, (**f**) HPH 100 group, (**g**) HPH 200 group, (**h**) HPH 300 group. All images are magnified 40×. CFA, complete Freund’s adjuvant; PDRN, polydeoxyribonucleotide; HPH, human placenta hydrolysate. *n* = 6 per group. Data are expressed as mean ± SD. **** *p* < 0.0001 vs. control, #### *p* < 0.0001 vs. CFA, analyzed using one-way analysis of variance followed by Tukey’s multiple comparisons.

The paw tissues of mice in each group were stained with hematoxylin & eosin (H&E) to observe the pathological changes (Figure 3A). As a result, the level of inflammation in the CFA group was clearly increased compared to the control group. The 20% glucose, PDRN, and HPH groups showed a significant decrease in inflammatory cell infiltration and a significant decrease in the number of neutrophils compared to the CFA group. The inflammation score results are shown in Figure 3B.

### 2.4. mRNA Expression of Pro-Inflammatory Cytokine Using qPCR in Spinal Cord and Levels of Cytokine of Serum

In central nervous system sensitization, nociceptive nerve cells can become sensitized due to damage or inflammation in peripheral tissues, which is believed to be a primary mechanism leading to chronic pain. Therefore, the analgesic mechanism of HPH was evaluated in paw tissue and analyzed primarily for spinal mechanisms, which involve the central nervous system [38].

**Figure 4:**
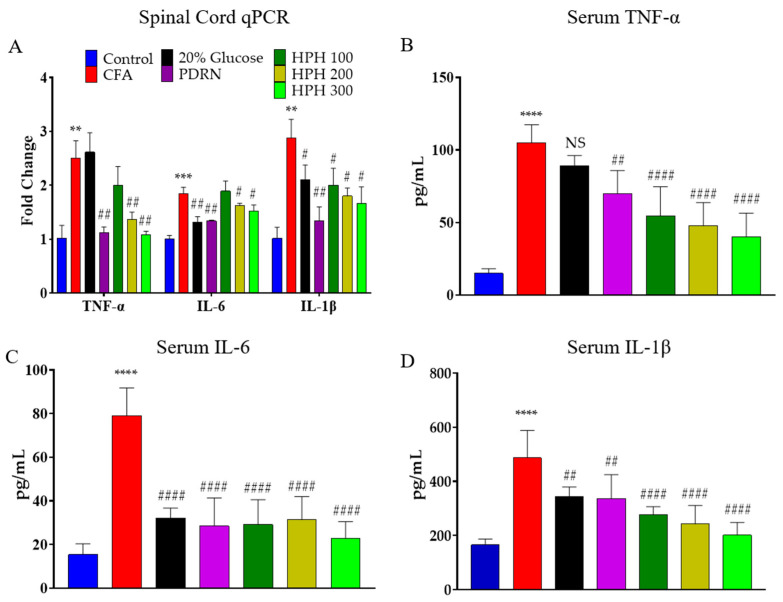
Effect of 20% Glucose, PDRN, and HPH on Cytokine Gene Expression in CFA-Induced Inflammatory Pain and Levels of Pro-Inflammatory Cytokines in Serum. Changes in gene expression of TNF-α, IL-6, and IL-1β in the spinal cord (**A**) and levels of cytokines in serum (**B**–**D**). CFA, complete Freund’s adjuvant; PDRN, polydeoxyribonucleotide; HPH, human placenta hydrolysate. *n* = 6 per group. Data are expressed as mean ± SD. NS, not significant. **** *p* < 0.0001, *** *p* < 0.001, ** *p* < 0.01 vs. control; # *p* < 0.05, ## *p* < 0.01, #### *p* < 0.0001 vs. CFA, analyzed using one-way analysis of variance followed by Tukey’s multiple comparisons.

Pro-inflammatory cytokine gene expression was assessed in the lumbar 4-5 sections of the spinal cord from CFA-treated mice. In the CFA-injected group, the expression levels of TNF-α, IL-6, and IL-1β were significantly higher compared to the control group (Figure 4A). The inhibition of TNF-α expression was significantly reduced in all groups except for the 20% glucose and HPH 100 group, while IL-6 expression inhibition was significantly reduced in all groups except for the HPH 100 group. IL-1β expression was significantly suppressed in all drug administration groups.

The serum levels of TNF-α, IL-6, and IL-1β in the CFA group were significantly higher than in the control group, indicating successful induction of inflammation. Upon quantification, serum TNF-α levels were significantly decreased in all drug-treated groups except for the 20% glucose. Quantitative analysis showed that IL-6 and IL-1β levels were significantly reduced in all five drug-treated groups (Figure 4B–D).

### 2.5. Glial Cell Activation

**Figure 5:**
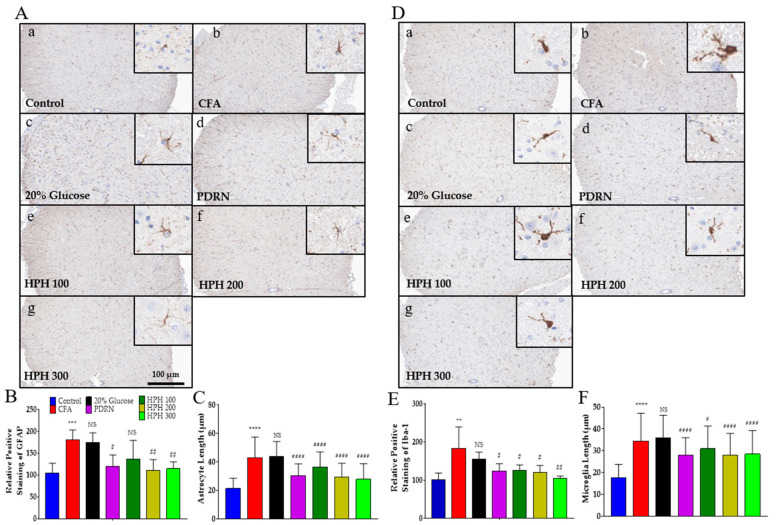
Immunohistochemical staining for GFAP and Iba-1 expression in the spinal cord and quantitative analysis of the expression levels. GFAP (**A**) and Iba-1 (**D**) expression in the spinal cord is indicated by dark brown staining, representing positive expression. The intensity of astrocytes and microglia (**B**,**E**) and the cell length (**C**,**F**) were analyzed using the ImageJ program version 1.54f, respectively. All images were magnified 100×. Panels: (**a**) control group, (**b**) CFA group, (**c**) 20% glucose group, (**d**) PDRN group, (**e**) HPH 100 group, (**f**) HPH 200 group, and (**g**) HPH 300 group. CFA, complete Freund’s adjuvant; PDRN, polydeoxyribonucleotide; HPH, human placenta hydrolysate. *n* = 6 per group. Data are expressed as mean ± SD. NS, not significant. **** *p* < 0.0001, *** *p* < 0.001, ** *p* < 0.01 vs. control; # *p* < 0.05, ## *p* < 0.01, #### *p* < 0.0001 vs. CFA, analyzed using one-way analysis of variance followed by Tukey’s multiple comparisons.

Glial cells primarily contribute to hypersensitivity and chronic pain, and astrocytes and microglia in the spinal cord play an important role in the development of hyperalgesia and allodynia in acute and chronic pain [39]. The histological examination of the spinal tissue from the CFA injection showed an increase in the immunolabeling of glial markers, both for microglia and astrocytes, in comparison to the control group (Figure 5A,C). Glial fibrillary acidic protein (GFAP), an astrocyte marker, significantly increased due to the CFA injection but was significantly decreased by the administration of PDRN, HPH 200, and HPH 300 (Figure 5B). Ionized calcium-binding adaptor molecule 1(Iba-1), a microglia marker, was also significantly increased by CFA injection and significantly decreased by PDRN and HPH administration (Figure 5D). Moreover, stellate astrocytes and microglia amoeboid morphology were observed to be more differentiated in the CFA group, demonstrating the presence of an inflammatory process.

### 2.6. Protein Expression of Pro-Inflammatory Cytokines Using Western Blot in Spinal Cord

**Figure 6:**
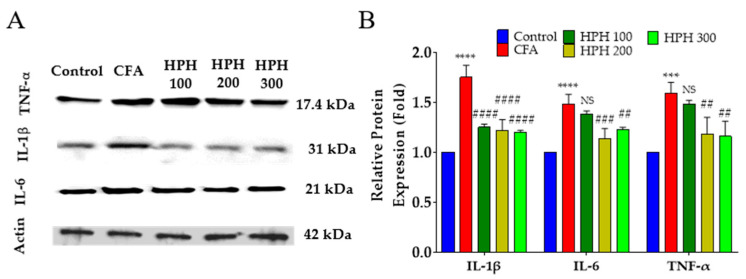
Effect of HPH on cytokine protein expression in the spinal cord in CFA-induced inflammatory pain. A representative protein analysis image of pro-inflammatory cytokines (**A**), and analyzed relative quantitative of TNF-α, IL-6, and IL-β protein (**B**). CFA, complete Freund’s adjuvant; HPH, human placenta hydrolysate, *n* = 6 in each group. Data are expressed as the mean ± SD. NS, not significant. **** *p* < 0.0001, *** *p* < 0.001 vs. control, ## *p* < 0.01, ### *p* < 0.001, #### *p* < 0.0001 vs. CFA with one-way analysis of variance followed by Tukey’s multiple comparisons.

The effect of HPH administration on pro-inflammatory cytokine protein expression in CFA-injected mice was assessed in the spinal cord. In the CFA injection group, protein expression levels of TNF-α, IL-6, and IL-1β were significantly increased compared to the control group (Figure 6A,B). TNF-α expression was significantly reduced in all groups administered HPH, while IL-6 expression was significantly reduced in the HPH 200 and HPH 300 groups but not in the HPH 100 group. IL-1β protein expression was significantly reduced only in the HPH 200 and HPH 300 groups.

In response to injury or inflammation, astrocytes and microglia become activated and contribute to the maintenance and amplification of pain through the release of pro-inflammatory cytokines, including TNF-α, IL-6, and IL-1β. As shown in the previous results, activated astrocytes and microglia release these cytokines, which can further sensitize neurons, thereby perpetuating pain signals.

The reduction in pro-inflammatory cytokine expression following HPH administration suggests a potential mechanism through which HPH alleviates pain. By modulating the activity of astrocytes and microglia, HPH may reduce the production and release of cytokines that are critical in the inflammatory response and pain signaling pathways. This modulation may lead to decreased neuronal sensitization and, consequently, a reduction in pain perception.

### 2.7. Decreased Expression of Phosphorylated Akt in the Spinal Cord after HPH Administration

Phosphorylated Akt (pAkt) generally regulates cell survival and growth, but it has recently been reported that this pathway is closely related to the development of chronic pain [40]. Elevated levels of pAkt have been observed in various pain conditions, suggesting that Akt phosphorylation may contribute to pain signal transduction and maintenance. Additionally, pAkt is known to be upregulated upon administration of the chemotherapy drug paclitaxel, which is associated with neuropathic pain [41].

**Figure 7:**
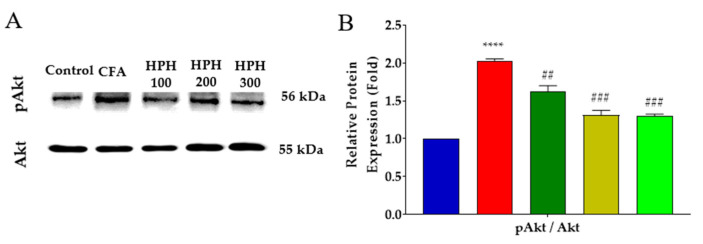
Effect of HPH on Akt and pAkt protein expressions in the spinal cord in CFA-induced inflammatory pain. A representative protein analysis image (**A**) and analyzed relative quantitative of Akt and pAkt proteins (**B**). CFA, complete Freund’s adjuvant; HPH, human placenta hydrolysate, *n* = 6 in each group. Data are expressed as the mean ± SD. **** *p* < 0.0001 vs. control, ## *p* < 0.01, ### *p* < 0.001 vs. CFA with one-way analysis of variance followed by Tukey’s multiple comparisons.

We analyzed the protein expression of Akt, a downstream factor significantly influenced by pro-inflammatory cytokines. In the CFA group, pAkt levels were significantly increased, indicating heightened Akt pathway activity potentially contributing to the pain state. However, phosphorylation of Akt was shown to be inhibited in all HPH administration groups (Figure 7A,B), suggesting that HPH may exert its antinociceptive effects partly by modulating the Akt pathway. This inhibition of Akt phosphorylation may reduce pain signal amplification in the spinal cord, thereby contributing to the observed pain relief.

## 3. Discussion

In this study, we investigated the antinociceptive effects of human placenta hydrolysate (HPH) on CFA-induced inflammatory pain and explored potential underlying mechanisms. HPH 100, 200, and 300 are groups that were injected intraperitoneally with human placental hydrolysate in mice at doses of 100, 200, and 300 μL, respectively. These doses are equivalent to approximately 2, 4, and 6 mL/kg when converted to human doses. Our results demonstrated that HPH alleviated CFA-induced edema and hypersensitivity. Additionally, prevention by HPH administration inhibited the expression of pro-inflammatory cytokines TNF-α, IL-6, and IL-1β in the spinal cord following CFA injection. Elevated levels of TNF-α, IL-6, and IL-1β were observed in the serum of CFA-injected mice, which were reduced after HPH administration.

Previous studies reported that HPH suppresses immune responses, improves healing of damaged ligaments, reduces degenerative changes, and promotes regeneration by increasing the expression of collagen type I and scleraxis (Scx) [42]. However, this study is the first to report the role of HPH in chronic pain in relation to inflammatory cytokines and glial cells. We injected CFA at the start and again on D14 (to induce a chronic animal model of inflammation) over a 28-day experimental period and demonstrated that repeated intraperitoneal injections of HPH could dose-dependently prevent the development of CFA-induced cold and mechanical allodynia. Moreover, HPH administration showed an anti-inflammatory effect equivalent to or better than 20% glucose or PDRN, which are anti-inflammatory agents currently used in clinical patients. HPH suppressed cold allodynia in a dose-dependent manner, and overall, HPH showed a higher antinociceptive effect on mechanical allodynia than on cold allodynia. We cannot explain the details of these results, but they may have resulted from the difference in the mechanism of cold and mechanical allodynia. Several studies have reported that at the spinal cord level, cold allodynia is mediated mainly by C fibers, while mechanical allodynia is transmitted by Aδ afferent fibers. The human placenta is important and plays a variety of roles, so its actual composition is complex and contains many substances. It contains vitamins, nucleic acids, minerals, amino acids, and growth factors [43]. It is expected that some substances among the HPH components suppressed inflammation, but it is not known exactly at present. We did not check the cytokine levels in the early or middle stages of inflammatory pain caused by CFA, but since the difference in edema was the greatest on D14, it is thought that there will be significant differences in the middle stages as well. In particular, pain is transmitted to the spinal cord and neurons of the central nervous system and plays a very important role in pain transmission and suppression. In this study, we confirmed that the cytokines, Akt, astrocytes, and microglia in the spinal cord were all significantly reduced due to HPH. We presume that among the HPH components, low-molecular substances that can pass through the BBB showed the main analgesic effect. Additional studies are needed to select individual components and study the mechanism of action.

During the transmission of neuropathic pain to the central nervous system, there are neurophysiological phenomena that can contribute to various clinical pain conditions [44]. Central nervous sensitization can cause pain due to the neural plasticity of central nervous system neurons, and it has been reported that multiple mechanisms, not a single mechanism, are involved in causing central nervous sensitization [45]. Ultimately, in central nervous sensitization, nociceptive neurons in the dorsal horn may become sensitized due to peripheral tissue damage or inflammation, and this is considered to be the main mechanism of central nervous sensitization causing chronic pain states, and these results were supported by animal studies and clinical studies [38]. Therefore, the analgesic mechanism of this HPH was limited to research on the peripheral nerves, which are influenced by various factors, and the analysis was focused on the spinal cord, which is the main mechanism of chronic pain. This study also analyzed peripheral nerve pathways, such as edema and pro-inflammatory cytokines mRNA analysis in mouse paw, but the main research analysis mainly focused on the central nerve system, the spinal cord. This may indicate that HPH’s signaling pathway or target molecules play a more significant role in mechanical pain processing. Furthermore, HPH injections significantly reduced CFA-induced paw edema. Overall, these results support that HPH has antinociceptive effects.

PDRN is DNA with a molecular weight of 50 to 1500 kDa isolated from sperm cells of Oncorhynchus mykiss (salmon trout) or Oncorhynchus keta (Chum Salmon) [46]. PDRN is known to have many positive therapeutic effects, including increased collagen synthesis [47], improved angiogenesis [48,49], anti-inflammation [33], and osteoblast activation [50]. PDRN activates adenosine A_2A_ receptor to regulate the cytokine network and alleviate ROS-related inflammatory diseases [51,52] and exhibits whitening effects by inhibiting tyrosinase-related protein-1 (TRP-1) and melanocyte-inducing transcription factor (MITF) [53]. In addition, PDRN has been reported to have beneficial effects on maintaining skin elasticity by inhibiting matrix metalloproteinases (MMP-1) and elastase expression [54].

The Akt signaling pathway is the most important protein in cell survival and is responsible for the signal cascade that transmits receptor stimulation under the cell membrane [55]. pAkt is generally referred to as a marker of PI3K activation, and PI3K and Akt signaling pathways may contribute to the development of neuropathic pain in the early stage [40]. Several lines of evidence have shown that intradermal capsaicin injection in rats causes hyperalgesia with Akt activation, and pain behavior can be mediated through the Akt signaling pathway [56]. In addition, in a paclitaxel-induced neuropathy model, cold and mechanical allodynia were attenuated when LY-294002 or MK-2206, and Akt inhibitor was administered intrathecally [41].

HPH consists of 95% water and 4.9% crude protein. The crude protein contains 15 kinds of amino acids, and the total content is more than 11 mg. In particular, aspartic acid, glycine, leucine, and lysine are contained in a high content of more than 1 mg/mL. Mineral content analysis was performed using ICP-MS. The analysis revealed significant levels of minerals commonly found in the human body, including Sodium (Na), Potassium (K), Magnesium (Mg), Calcium (Ca), and Phosphorus (P). Among these, we are interested in leucine, and we would like to propose a hypothesis on the relationship between leucine and pAkt. According to studies on leucine and pAkt, when leucine is removed from the medium in HEK293 cells, phosphorylation of Akt, 4EB-P1, and S6K1 is promoted, suggesting that a diet without leucine is not effective for growth-related diseases [57]. In addition, it has been reported that leucine increases amylase synthesis in pancreatic cells and decreases phosphorylation of Akt, PI3K, S6K1, and mTOR [58].

In this study, we further confirmed that protein expression of pAkt increased in the spinal cord after CFA injection. This is consistent with the report that pAkt expression in neurons is upregulated after injection of paclitaxel, a chemotherapy drug [41]. Intraplantar injection of CFA-induced chronic pain in the spinal cord and increased pAkt expression, which was suppressed by HPH administration, these results suggest that the antinociceptive effect of HPH may be mediated by central sensitization through pAkt in the spinal cord. In placenta extract studies, porcine placenta extract was found to be beneficial in the prevention and treatment of osteoporosis through regulation of ERK 1/2 and Akt in human osteoblast hFOB 1.19 cells [59], and the role of porcine placenta extract established in influencing the correlation between ceramide synthase 3 and Akt in HaCaT cells was defined, but this study is the first to report on the relationship between human placenta and Akt [60].

When microglia and astrocytes are activated, they release a variety of analgesic substances, and inflammatory cytokines have been identified as mediators of hyperalgesia and pain [61,62]. Additionally, as a result of CFA injection into the mouse plantar, astrocytes and microglia were upregulated in the subacute and chronic stages, and glial cell products TNF-a, IL-6, and IL-1 β were increased at the mRNA and protein levels [63]. Previous reports and this HPH study suggest that CFA-induced peripheral inflammation is induced by robust glial activation in the spinal cord, which induces inflammatory cytokines. In addition, similar to the hypersensitive glial cell activation, it precedes CFA-induced peripheral neuropathy and inflammation, supporting a consistent theory that enhanced cytokine expression in CNS-induced hypersensitivity. Meanwhile, since glycyrrhizin reduces pro-inflammatory cytokines through glial cells, inhibiting inflammatory pain and suppressing peripheral pain [64], it is expected that HPH also exhibits cytokine reduction and analgesic effects through inhibition at the glial cell expression levels.

IL-1β, IL-6, and TNF-α are well-known pro-inflammatory cytokines associated with acute and chronic inflammatory pain [65]. TNF-α was revealed in the majority of voltage-gated sodium channel 1.3-positive neurons and is traditionally considered the major isoform expressed in the brain and spinal cord [66]. Pain generation and sensitization by spinal IL-1β are consistent with previous studies showing induction of long-term potentiation of the spinal nociceptive pathway in spinal IL-1β, which shares a pro-inflammatory central sensitization process [67,68,69]. Additionally, spinal IL-1β has been found to be increased after carrageenan administration [70], intraplantar injections of zymosan or formalin [71], and during the development of chronic arthritis, as evidence that inflammation elevates spinal IL-1β [72]. IL-6 is a pleiotropic cytokine and is related to many functions throughout the body [73]. In the central nervous system, IL-6 plays an important role in regulating neurotransmitter biosynthesis [74], promoting neuronal survival [75], stimulating astrocyte proliferation [76,77,78], inducing neuronal differentiation [79], and regulating pain [80,81]. The anti-inflammatory and antinociceptive effects of HPH may have different compensatory effects due to different mechanisms. Collectively, these findings suggest that HPH may exert its antinociceptive effects by inhibiting Akt-TNF-α/IL-6/IL-1β signaling.

Glial activation is a common feature of central nervous system diseases [82], and there is increasing evidence that it plays an active role in neuroprotection or neurodegeneration [83]. Recently, microglia and astrocytes in the spinal cord have been recognized as having a potential role in the initiation and maintenance of pain caused by inflammation, peripheral nerves, and spinal nerve damage [71,84]. Microglia play a direct role in pain generation, and microglia cells showed analgesia through downregulation of the CXCR7/PI3K/Akt signaling pathway in chronic post-surgical pain [85]. Consistently, HPH also showed analgesic effects through microglia cells and pAkt inhibition. Discoveries about the role of glial cells in the pathophysiological processes of acute and chronic pain conditions provide promising alternatives for new pain treatment strategies [39]. It is generally known that glial cells mainly contribute to hypersensitivity in chronic pain. However, astrocytes and microglia in the spinal cord play an important role in the development of hyperalgesia and allodynia in acute and chronic pain. Studies of different types of pain and astrocyte/microglia suggest that they may share a common pathophysiological mechanisms and that these may be present in spinal cord immune responses. In addition, as reported in this study, overexpressed astrocyte and microglia indicate pain, and the results of HPH regulating them to provide antinociception are consistent with previous studies. Overall, HPH showed analgesic effects by altering protein expressions related to inflammatory factors and neuronal improvement in the CFA-induced inflammatory and neuropathic pain model, suggesting that HPH is an effective drug candidate for inflammatory and neuropathic pain.

## 4. Materials and Methods

### 4.1. Chemicals

Complete Freund’s Adjuvant (CFA) was purchased from Sigma Chemical. 20% glucose and polydeoxynucleotide (PDRN) were obtained as injectable solutions from JW Joongwe Pharmaceutical (Seoul, Republic of Korea) and MASTELL pharmaceutical company (Sanremo, Italy), respectively. HPH is Laennec, a hydrolysate of human placenta extract, and was supplied by GC Green Cross (Yong-in, Republic of Korea).

### 4.2. Animals

Male C57BL6J mice aged 6 weeks obtained from DBL (Daehan Bio, Chungbuk, Republic of Korea) were nourished in temperature-controlled conditions on 12 h light/dark cycle with water ad libitum. The experimental protocol followed the guidelines recommended by the Animal Care and Committee hosted by Kyung-Hee University. The permission number of animal ethics is KHUASP-24-411(20 May 2024). There are 45 mice; each group contains 6–7 mice. All behavioral assessments were conducted during the same time period.

### 4.3. Establishment of an Inflammatory Pain Model Using CFA Injection

To establish a mice inflammatory pain model, the mice were lightly anesthetized using isoflurane, and CFA was injected into the plantar surface of the right hind paw [86,87]. The CFA administration groups were injected 20 μL CFA into the right hind paw of the mice, and the control group was injected with saline solution at the same site. Mice were divided into a control group, a CFA group, and a CFA + drug administration group. Seven groups of mice were administered vehicle (100 μL/mouse) or 20% glucose (100 μL/mouse), PDRN (100 μL/mouse), HPH 100 (100 μL/mouse), HPH 200 (200 μL/mouse), HPH 300 (300 μL/mouse) was administered. The experiment period lasted a total of 28 days (D28), and CFA was additionally administered to the hind paw on D14 for an inflammation-boosting effect. Drugs were administered twice a week, and behavior was evaluated once a week.

### 4.4. Behavioral Assessment

All behavioral tests were performed as specified in previous studies [88,89]. Cold and mechanical allodynia were measured using acetone and von-Frey filament test, respectively. Mice trapped in cages were evaluated by placing them on a wire mesh dedicated to behavioral experiments, and they were allowed to acclimate to the wire mesh for 30 min before all behavioral tests. For cold allodynia and quantification, acetone (10 μL) was applied to the center of the hind paw of the mice. Pain responses (licks and flicks) to acetone were observed and counted for 30 s. Therefore, the term “# of Response” mentioned in the resulting figures refers to the average number of pain responses per acetone.

The von Frey method was used to measure mechanical allodynia. After 30 min of acclimation, responses were elicited by stimulating the hind paw of the mice using a von Frey filament (Stoelting, WI, USA). Filaments with different stiffness (0.02, 0.04, 0.07, 0.16, 0.4, 0.6, 1, 1.4, and 2 g) were stimulated to the hind paw. The “50% threshold” was calculated according to the Chaplan’s and Dixon’s calculations [90,91]. After the evaluation was completed, the mice were euthanized with isoflurane. Blood was collected from the left ventricle of the mice, and then intracardiac perfusion was performed with iced PBS. Tissue sampling for analysis was collected from the lumbar 4–5 spinal segments of the spinal cord and the right foot (up to the ankle).

To verify the peripheral inflammatory edema of the mice paw, the thickness was measured using a caliper. Calipers measured paw thickness in millimeters along the dorsal-plantar axis at the level of the metatarsals. Measurements were taken twice a week during the experimental period.

### 4.5. Gene Expression Using Gene-Specific Primers

Total ribonucleic acid (RNA) was isolated from spinal cord and mice paw tissue samples by an AccuPrep RNA kit (Bioneer, Daejeon, Republic of Korea) according to the user’s protocol. RNA levels were estimated NanoDrop (Thermo, Middlesex, MA, USA). RNA (0.5 μg) was transcribed into complementary DNA by Maxime RT Premix (Intronbi, Seongnam, Republic of Korea). Then the single-stranded cDNA was used as a template in qRT-PCR, which was performed by using a SYBR kit (Merdian, Cincinnati, OH, USA) and the Real-Time thermocycler (Bio-Rad, Hercules, CA, USA). The target genes of the primers used are mouse IL-6, IL-1β, and TNF-α, and the housekeeping is GAPDH. The primer are as follows: The sense primer of 5′-GGAGAGGAGACTTCACAG-3′ and antisense primer of 5′-GCCATTGCACAACTCTTTTTC-3′ for IL-6; The sense primer of 5′-CAACCAACAAGTGATATTCTCCATG-3′ and antisense primer of 5′-GATCCACACTCTCCAGCTGCA-3′ for IL-1β; The sense primer of 5′-CATCTTCTCAAAATTCGAGTGACAA-3′ and antisense primer of 5′-TGGGAGTAGACAAGGTACAACCC-3′ for TNF-α; The sense primer of 5′-GTCGTGGAGTCTACTGGTGTCTTC-3′ and antisense primer of 5′-GTCATCATACTTGGCAGGTTTCTC-3′ for GAPDH. The PCR condition was as follows: initial denaturation at 94 °C for 10 min and then 94 °C for 20 s, 58 °C for 20 s, and 72 °C for 20 s for 40 cycles. Threshold Ct and cycle were measured, as samples correlate inversely with the messenger RNA. The relative quantities of gene expression, fold change to the housekeeping gene GAPDH, was calculated according to 2^−∆∆Ct^ method, and the expression value of the control was expressed as 1.

### 4.6. Western Blot

Spinal cord tissues were homogenized using radio-immunoprecipitation assay buffer and centrifuged at 12,000 rpm for 10 min. Bradford quantification (Bio-Rad Laboratories, Hercules, CA, USA) determined protein concentrations. Equal amounts of protein contents (30 μg) were separated by 12% sodium dodecyl sulfate-polyacrylamide gel electrophoresis and transferred onto a nitrocellulose membrane at 120 V for 110 min. The membranes were blocked with 5% non-fat milk for 1 h at room temperature, followed by incubation with primary antibody overnight at 4 °C. The specific primary antibodies used included anti-Akt (1:2000, #NBP3-21578, Novus Biologicals, Littleton, CO, USA), anti-pAkt (1:1000, #700392, Waltham, MA, USA), anti-IL-6 (1:500 dilution, #NB600-1131, Novus, Littleton, CO, USA), anti-IL-1β (1:500 dilution, #NB600-633, Novus, Littleton, CO, USA), anti-TNF-α (1:500 dilution, #NBP1-19532, Novus, Littleton, CO, USA) and anti-actin (1:1000 dilution, #NB100-1617, Novus, Littleton, CO, USA). The membranes were washed three times with TBS-T and then incubated with HRP conjugated anti-rabbit secondary antibody (1:1000 dilution, #31460, Thermo, Waltham, MA, USA) in 5% skim milk at room temperature for 1 h. The bounded antibodies were developed using an enhanced chemiluminescence solution (D-Plus system, Seoul, Republic of Korea).

### 4.7. Enzyme-Linked Immunosorbent Assay (ELISA)

Serum samples from all groups (Control, CFA, 20% glucose, PDRN, and 3 concentrations of HPH) were analyzed using ELISA kits. IL-6, TNF-α, and IL-1β ELISA kits (R&D system, Minneapolis, MN, USA) were used following the user’s protocol. The spinal cord was extracted, and tissue protein insolation was performed using cold PBS to a final concentration of 100 mg of protein using RIPA. The sample grinds were then centrifuged (4 °C, 12,000 rpm, 10 min). The supernatant was analyzed according to the kit user’s protocols.

### 4.8. Hematoxylin and Eosin Staining and Inflammation Scoring

Mice paw tissues were isolated and fixed in 10% paraformaldehyde for 48 h. Water was removed by dipping the paws in gradient alcohol concentrations, and then the paws were embedded in paraffin. Paraffin blocks were sliced, stained with hematoxylin and eosin, and observed using a microscope.

The level of inflammation in paw tissues was quantified based on a 0–5 point scoring system. The inflammation score was defined and scored as follows: 5 = severe inflammation, 4 = moderate/severe inflammation, 3 = moderate inflammation, 2 = mild/moderate inflammation, 1 = mild inflammation, and 0 = no inflammation [92].

### 4.9. Immunohistochemistry Analysis

Mice were anesthetized with isoflurane and perfused intracardially with 20 mL PBS, followed by 20 mL fixative 4% paraformaldehyde. L4–L5 segments of spinal cords are extracted, fixed in paraformaldehyde, and embedded in paraffin. Sections of 10 μm were de-paraffinized and dewatered by sequential processes with xylene and alcohol and then washed with PBS. Antigen retrieval was processed by immersion of slides in citrate buffer and boiling for 30 min, followed by cooling of the slides for 30 min. Endogenous peroxidase was quenched using H_2_O_2_ (3% in PBS) for 10 min and blocked with 2.5% normal horse serum for 30 min to block unspecific binding. The slides were incubated with two primary antibodies, GFAP (1:50,000 dilution, #NBP300-141, Novus Biologicals, Littleton, CO, USA) and Iba-1 (1:20,000 dilution, #NBP100-1028, Novus Biologicals, Littleton, CO, USA), respectively overnight at 4 °C. The next day, the slides were incubated with HRP (horse radish peroxidase)-conjugated IgG polymer reagent (Vector Laboratories, Burlingame, CA, USA), a secondary antibody, at room temperature for 1 h. The colorimetric reaction was visualized using the 3,3′-diaminobenzide tetrahydrochloride (DAB) kit (Vector Laboratories, Burlingame, CA, USA). Afterward, the slides were washed with distilled water and counterstained with Harris hematoxylin. All IHC-stained slides were scanned with an Aperio Scan Scope (Leica Biosystems, Buffalo Grove, IL, USA). The size of astrocytes and microglia was analyzed using the ruler toolbar in the Aperio Imagescope v12.4 (Leica Biosystems) program.

### 4.10. Statistical Analysis

The statistical analyses were performed using GraphPad Prism version 9.0 (GraphPad Software Inc., San Diego, CA, USA). Data are expressed as mean ± standard deviation. Two-way analysis of variance, followed by Tucky’s post-tests for multiple comparisons for behavioral tests. The distribution normality was tested using the D’Agostino-Pearson omnibus test. In addition, statistical analyses were conducted using one-way ANOVA followed by Tukey’s test for IHC quantification, protein, and gene expression. A level of *p* < 0.05 was regarded as significant.

## 5. Conclusions

Our study showed that intraperitoneal injection with HPH significantly reduced CFA-induced pain and decreased IL-1β, TNF-α, and IL-6 and inhibited pAkt upregulation in the spinal cord. These findings suggest that HPH can attenuate chronic inflammatory pain through inhibition of the TNF-α-IL-6-IL-1β/pAkt signaling in the spinal cord. Our research provided scientific data for preclinical and clinical studies of HPH for the prevention of chronic inflammatory pain.

## Data Availability

All the data supporting the conclusions of this study are included in the manuscript.
